# Removal of dental implants displaced into the maxillary sinus: a retrospective single-center study

**DOI:** 10.1186/s13005-022-00339-w

**Published:** 2022-11-17

**Authors:** Francesco Bennardo, Selene Barone, Caterina Buffone, Walter Colangeli, Alessandro Antonelli, Amerigo Giudice

**Affiliations:** 1 School of Dentistry, Department of Health Sciences, Magna Graecia University of Catanzaro, Viale Europa, 88100 Catanzaro, Italy; 2 Unit of Maxillofacial Surgery, Academic Hospital of Magna Graecia University of Catanzaro, Catanzaro, Italy

**Keywords:** Dental Implants, Foreign-Body Migration, Maxillary Sinusitis, Sinus Floor Augmentation

## Abstract

**Background:**

The use of dental implants in the prosthetic rehabilitation of the posterior atrophic maxilla might be a challenge procedure because of low bone quantity and quality. This study aimed to report cases of implant displacement or migration into the maxillary sinus treated from 2008 to 2021.

**Materials and methods:**

All patients with unintentional insertion and/or displacement of dental implants into the maxillary sinus cavity that underwent surgical removal were included. Variables assessed included the patients’ characteristics, past medical history, clinical and radiological findings at presentation, surgical approach (transoral, transnasal, combined), and outcome.

**Results:**

A total of forty patients (23 male, 17 female) underwent surgical removal of dental implant displaced in the maxillary sinus. The mean age was 52,3 + 11,3 years. Seven patients presented with oro-antral fistula (OAF). In 15 cases, an ostium obstruction was diagnosed. Twenty-five patients underwent transoral surgery under local anesthesia. Eleven patients were treated solely via transnasal endoscopic approach, and four patients who had an associated OAF underwent surgery through a combined transnasal and transoral approach. All patients healed uneventfully without complications.

**Conclusion:**

These results and recent literature validate that transoral and transnasal approach, or a combination of these procedures, can be used safely to treat complications following displacement/migration of dental implants in the maxillary sinus. Early surgical removal minimizes sinus inflammation and prevents more invasive procedures. Each procedure presents specific indications that must be carefully evaluated prior to treatment choice to optimize intervention outcomes.

## Introduction

Titanium dental implants have been used worldwide for almost half a century with a low failure rate. Bone quality and quantity could influence fixture osseointegration and the success of implant-prosthetic procedures. Using dental implants in the prosthetic rehabilitation of the posterior atrophic maxilla is a standard procedure. However, it might be a challenge because of low bone density, insufficient bone height due to progressive resorption of the alveolar ridge, and pneumatization of the maxillary sinus [1–4].

The mechanism of bone resorption and sinus pneumatization are not yet well known: among the hypotheses, there is the lack of functional forces related to the functioning of the dental elements, tooth loss, and the subsequent alteration of the physiological process of bone resorption/repositioning, also because of positive air pressure into the sinus cavity as proved by previous human studies regarding the downward expansion of the maxillary sinus after dental extraction [5].

Sinus augmentation with lateral or transcrestal technique allowed implant placement in the posterior atrophic maxilla at the same surgical time or after 6–9 months. Short implants (length < 8 mm) can be used in specific cases to avoid these procedures and reduce morbidity and complications [6].

Implants placed in posterior maxillae contextually with sinus augmentation are associated with two main risk factors: a smoking habit of > 15 cigarettes/day and a residual ridge height of < 4 mm. These variables significantly affect implant survival rates and should be carefully evaluated by clinicians. Sinus floor elevation is a predictable procedure with low morbidity and a 90% implant survival rate. Graft infection was reported in about 5% of patients [7].

Insufficient primary stability of fixtures inserted close to or inside the maxillary sinus can lead to complications. These complications may include infection, osseointegration failure, bleeding, and migration [8]. Dental implant migration is defined as the displacement of a titanium fixture from its socket to a cavity or complex space of the facial region (including nasal and paranasal sinuses). The displacement could occur at the time of surgery or later. This condition can lead to infection and foreign body reaction. Migration into the paranasal sinuses can lead to sinusitis with marked symptoms and risks of disease progression [9].

The incidence of this complication remains unknown because of the lack of studies on this topic. Several articles concerning implant migration are in the literature, but most are case reports and case series describing a single approach (transoral, transnasal or combined) without analysing factors related to implant displacement.

This study aimed to report the analysis of forty cases of implant displacement or migration into the maxillary sinus treated in the Academic Hospital of Magna Graecia University of Catanzaro from January 2008 to December 2021.

## Materials and methods

The present article is reported according to the RECORD statement [10].

### Study design

According to the Declaration of Helsinki on medical protocol and ethics, the regional Ethical Review Board of Central Calabria (reference for the Magna Graecia University of Catanzaro) approved the study (n.171/2020). The study was designed as a retrospective observational single-center study.

### Study sample and data collection

All patients with unintentional insertion and/or displacement of dental implants into the maxillary sinus cavity that underwent or were candidates for surgical removal presenting between January 2008 to December 2021 at the Academic Hospital of Magna Graecia University of Catanzaro, Italy, were included in the study. The exclusion criteria were as follows: person under the age of 18; lack of three-dimensional radiographic images; treatment with any drug that may affect tissue healing; patients with immune system dysfunction or hematological disease; pregnancy or breastfeeding. Informed consent was obtained from all patients included in this study.

Study variables included the patients’ characteristics, past medical history, clinical findings at presentation, imaging findings at presentation, dentists’ certifications and information reported by patients regarding implant surgery, time-span from fixture insertion to presentation and surgical removal, findings at surgical removal, and outcome. The following outcome measures were considered:

1. clinical signs and symptoms: unilateral nasal obstruction, purulent nasal secretions, pain;
2. fixture and prosthetic components: number, type, shape, diameter, and length;
3. clinical conditions: site, sinus lift, time from implant placement, residual bone quantity at the original implant site (rounded to the nearest integer).

### Procedures

**Fig. 1:**
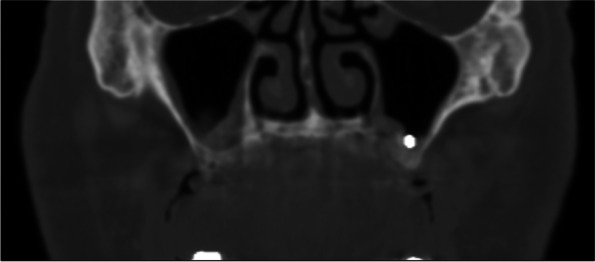
Displaced dental implant in the maxillary sinus with no evidence of ostium obstruction

**Fig. 2:**
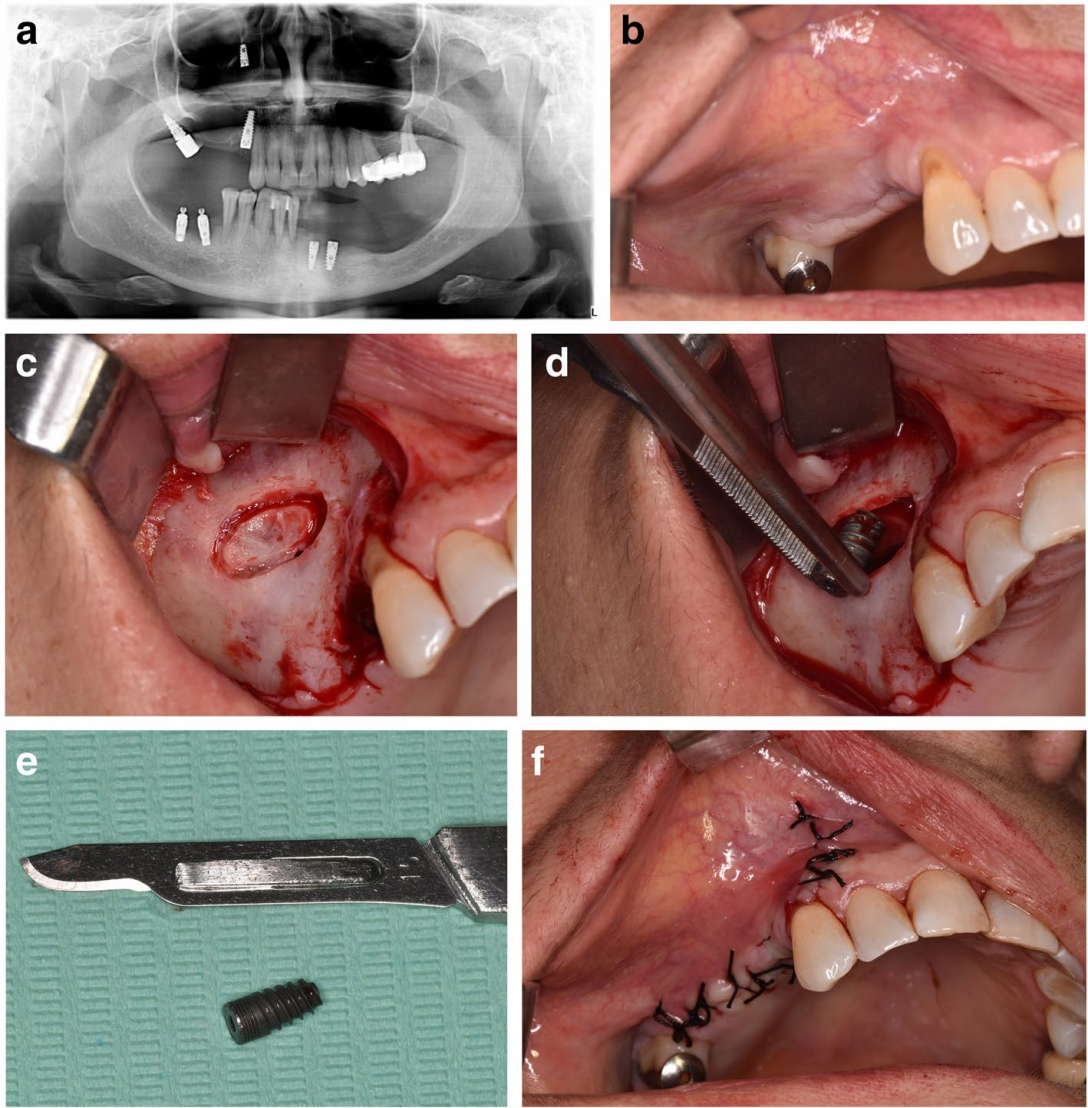
**A, B** Initial situation; **C** Mucoperiosteal flap elevation and lateral antrostomy; **D, E** Fixture removal; **F** Suture

**Fig. 3:**
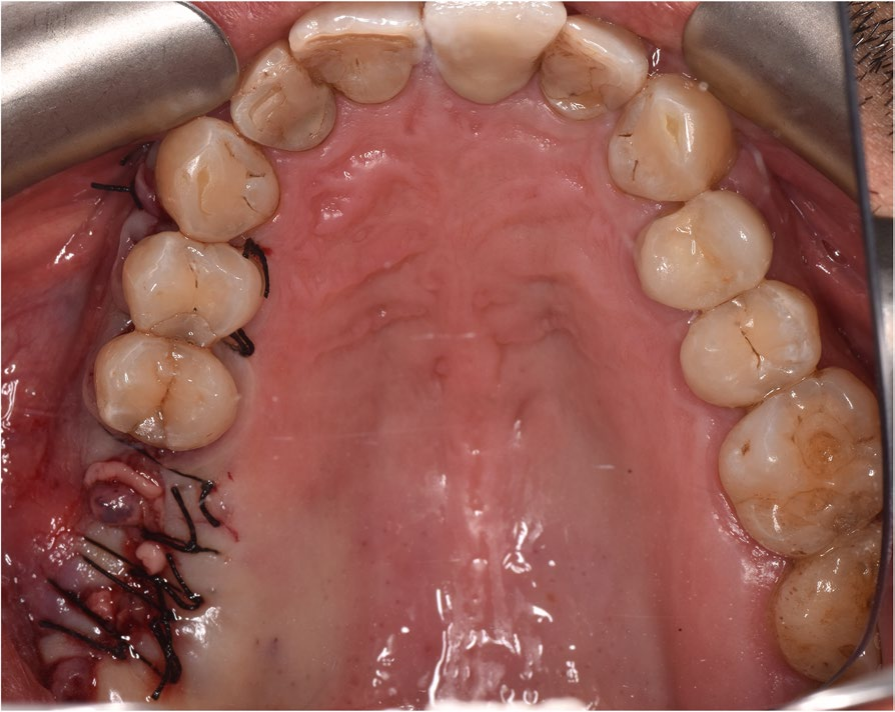
Closure of OAF by buccal flap (Rehrmann flap)

**Fig. 4:**
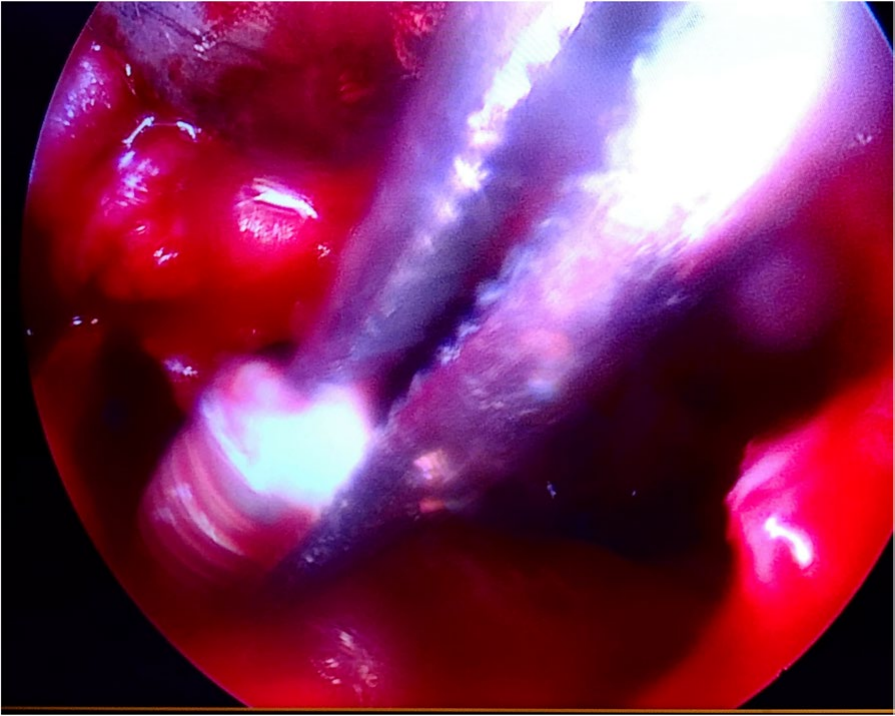
Endoscopic removal of dental implant

All the surgical procedures were performed by a certified oral and maxillofacial surgeon. Surgical removal of dental implants was performed under local/general anesthesia. A preoperative computed tomography scan was performed on all surgical candidates. A trans-oral approach was the only intervention in displaced dental implant cases, with or without sinusitis, but with no evidence of ostium obstruction (Fig. 1). The trans-oral approach comprised mucoperiosteal flap elevation and lateral antrostomy performed with diamond or tungsten carbide burs in a high-speed handpiece or piezoelectric device (Figs. 2 A-F). In the case of oroantral fistula (OAF), the implant could be removed through pre-existing communication. Then, after fistulectomy, the OAF was repaired using a mucoperiosteal flap with or without advancement of the buccal fat pad (Fig. 3). A trans-nasal approach was used whenever the displaced implant was associated with ostium obstruction (Fig. 4). The trans-nasal approach, always performed under general anesthesia, consisted of functional endoscopic sinus surgery (FESS).

FESS comprised uncinectomy and middle meatal antrostomy with maxillary sinus ostium enlargement to restore its adequate patency. In case of infection/inflammation of the other paranasal sinuses, the FESS allows to carry out the treatment simultaneously. A combined trans-nasal and trans-oral approach was used whenever the displaced implant was associated with ostium obstruction and OAF and/or alveolar bone infection or if the fixture could not be achieved via the middle meatus during FESS. In these cases, the surgery started with a trans-nasal approach to restore osteomeatal complex patency and assure sinus drainage. Then a trans-oral approach was used to retrieve the fixture and debride any inflammation of the alveolar process and close the communication. Patients received an antibiotic therapy with amoxicillin (1 g every 8 h) for ten days, beginning three days before surgery (clindamycin 600 mg 3 times a day, if allergic). Corticosteroid therapy with prednisone 25 mg (once daily for three days, beginning the day of surgery) was administered. Immediate postoperative pain control consisted of paracetamol. All patients were instructed to irrigate the nasal cavity with normal saline twice daily for the first two months and observe good oral hygiene. Follow-up was conducted at 1, 2, 6 months, and one year postoperatively. An oral examination was performed to check for dehiscence and signs of infection and inflammation at the repair site. Patients were asked to report any episode of sinusitis or need for medical therapy between the follow-up visits.

### Statistical analysis

Descriptive statistics recorded mean and standard deviation for continuous quantitative variables and absolute and relative frequencies for categorical data. The analysis of variance was performed using a two-tailed Student t-test. The level of significance was set at α = 0.05. Statistical analysis was performed by using the STATA software program (STATA Release 14; STATA Corporation, College Station, TX).

## Results

### Study population

Patients’ data are summarized in Table 1. From 2008 to 2021, forty patients underwent surgical removal of dental implant displaced in the maxillary sinus in the Academic Hospital of Magna Graecia University of Catanzaro. All the implants had been placed in private dental offices. Patients booked a hospital visit spontaneously or after a dental consultation. The mean age was 52,3 + 11,3 years. Twenty-three patients were male (57.5%), and 17 were female (42.5%), with a male to female ratio of 1.35:1. Fourteen patients were smokers (35%). Five patients had diabetes (12,5%).

**Table 1:** Patients’ data

Case	Gender	Age (years)	Diabetes	Smoking	Year of surgery	Number of fixture (site)	Other components	Fixture type	Fixture shape	Implant diameter x lenght (mm)	Time from placement to removal (months)	Residual bone height (mm)	Sinus lift	Sinusitis	OAF	Ostium obstruction	Other Signs/Symptoms	Surgical approach
1	F	45	No	Yes	2008	1 (16)	Healing abutment	TL	Cylindrical	3.3 × 10	12	3	No	Yes	No	Yes	Pain, nasal discharge	Transnasal
2	F	56	No	No	2008	2 (15. 17)	Cover screw	BL	Cylindrical	4.1 × 10; 4.8 × 10	4	6	Lateral	No	No	No	None	Transoral
3	F	61	Yes	No	2008	1 (16)	Cover screw	BL	Cylindrical	3.5 × 13	6	11	No	No	No	No	None	Transoral
4	M	50	No	Yes	2008	1 (17)	Abutment	BL	Conical	4.0 × 9	15	4	Crestal	Yes	Yes	Yes	Pain, nasal obstruction	Combined
5	M	38	No	No	2008	1 (26)	Cover screw	BL	Cylindrical	4.0 × 10	4	6	No	Yes	No	Yes	Nasal obstruction	Transnasal
6	F	42	No	No	2009	1 (27)	Abutment	BL	Cylindrical	4.0 × 10	4	5	No	No	No	No	None	Transoral
7	M	37	No	Yes	2009	1 (26)	Cover screw	BL	Cylindrical	5.0 × 9	6	4	Yes	Yes	No	No	Pain	Transoral
8	F	54	No	No	2009	1 (15)	Cover screw	BL	Conical	4.3 × 10	7	2	Yes	No	No	No	None	Transoral
9	F	60	No	No	2009	1 (26)	Cover screw	BL	Cylindrical	3.5 × 11	6	5	Crestal	Yes	Yes	Yes	Pain, nasal discharge	Combined
10	M	41	No	Yes	2009	2 (25, 27)	No	BL	Cylindrical	4.1 × 10; 4.1 × 8	7	6	Lateral	Yes	No	Yes	Nasal obstruction	Transnasal
11	M	52	No	Yes	2010	1 (17)	No	BL	Conical	3.75 × 11	4	3	Lateral	No	No	Yes	None	Transnasal
12	M	48	No	No	2010	1 (26)	Cover screw	BL	Cylindrical	4.0 × 12	4	4	No	No	No	No	None	Transoral
13	F	61	No	No	2010	1 (26)	No	BL	Cylindrical	4.0 × 9	1	4	No	No	No	No	None	Transoral
14	F	70	Yes	No	2010	1 (16)	Cover screw	BL	Cylindrical	4.3 × 11.5	4	4	No	No	No	No	None	Transoral
15	F	55	No	No	2011	1 (16)	Abutment	BL	Cylindrical	4.0 × 9	8	5	Crestal	Yes	Yes	Yes	Pain, nasal obstruction	Combined
16	F	50	No	No	2011	1 (26)	No	BL	Cylindrical	4.0 × 10	6	8	No	No	No	No	None	Transoral
17	F	55	No	No	2011	1 (26)	Cover screw	BL	Conical	4.0 × 11	26	6	Crestal	Yes	No	Yes	Pain, nasal obstruction, nasal discharge	Transnasal
18	M	46	No	No	2012	1 (16)	Healing abutment	TL	Cylindrical	4.8 × 8	20	2	No	Yes	No	Yes	Pain, nasal discharge, uveitis	Transnasal
19	M	49	No	Yes	2012	1 (26)	Cover screw	BL	Cylindrical	4.0 × 10	10	6	No	Yes	Yes	Yes	Pain, nasal obstruction, nasal discharge	Combined
20	F	65	No	No	2012	1 (16)	Cover screw	BL	Conical	4.0 × 10	8	3	No	No	No	Yes	None	Transnasal
21	M	39	No	Yes	2013	1 (16)	No	BL	Cylindrical	4.3 × 8.5	4	5	No	No	No	No	None	Transoral
22	M	45	No	No	2013	1 (16)	No	BL	Cylindrical	4.3 × 11.5	4	4	No	Yes	No	No	Pain, nasal discharge	Transoral
23	M	62	Yes	Yes	2013	1 (26)	No	BL	Cylindrical	4.0 × 8	7	4	No	No	No	No	None	Transoral
24	F	72	No	No	2014	1 (26)	No	BL	Conical	4.0 × 8	6	2	Crestal	No	No	No	None	Transoral
25	M	36	No	Yes	2014	1 (26)	Cover screw	BL	Conical	4.0 × 8	3	3	No	No	No	No	None	Transoral
26	M	83	Yes	No	2015	1 (26)	Abutment	BL	Conical	3.75 × 11.5	34	1	No	Yes	No	No	Pain	Transoral
27	F	48	No	No	2015	1 (26)	No	BL	Conical	4.0 × 8	3	3	Crestal	No	Yes	No	None	Transoral
28	F	53	No	No	2016	1 (16)	No	BL	Conical	4.0 × 11	5	3	No	No	No	No	None	Transoral
29	M	51	No	Yes	2016	1 (26)	Abutment	BL	Conical	3.5 × 10	6	2	No	Yes	No	Yes	Pain, nasal obstruction, nasal discharge	Transnasal
30	M	39	No	No	2017	1 (16)	Abutment	BL	Cylindrical	4.0 × 10	6	2	Crestal	Yes	No	Yes	Pain, nasal obstruction	Transnasal
31	M	41	No	No	2017	1 (26)	Abutment	BL	Conical	4.0 × 13	8	4	No	Yes	Yes	No	Pain	Transoral
32	F	40	No	No	2018	1 (16)	Cover screw	BL	Cylindrical	4.0 × 8	4	3	No	Yes	No	Yes	Pain, nasal obstruction, nasal discharge	Transnasal
33	M	66	No	Yes	2018	1 (26)	No	BL	Cylindrical	3.5 × 10	12	3	No	No	No	No	None	Transoral
34	M	49	No	No	2019	1 (16)	Cover screw	BL	Cylindrical	4.3 × 11.5	0.5	5	Lateral	No	No	No	None	Transoral
35	M	58	No	No	2019	1 (26)	Abutment	BL	Cylindrical	4.0 × 7	6	7	No	No	No	Yes	None	Transnasal
34	F	60	No	No	2020	1 (16)	Cover screw	BL	Conical	3.75 × 8	6	3	No	Yes	Yes	No	None	Transoral
35	M	42	No	Yes	2020	1 (26)	No	BL	Conical	4.0 × 10	14	3	No	No	No	No	None	Transoral
38	M	55	No	Yes	2021	1 (26)	Abutment	BL	Conical	4.0 × 9	12	1	No	No	No	No	None	Transoral
39	M	76	Yes	No	2021	1 (26)	Abutment	BL	Cylindrical	4.3 × 11.5	8	3	No	No	No	No	None	Transoral
40	M	42	No	Yes	2021	1 (16)	Healing abutment	TL	Cylindrical	4.1 × 4	0.5	4	No	No	No	No	None	Transoral

### Implants characteristics

The displaced implants were removed immediately after fixture insertion in three cases (7,5%), in the first six months of healing in 21 cases (52,5%), and after six months in 16 cases (40%). The most frequently involved site was the upper first molar area (34 cases; 85%). Two fixtures migrated into the maxillary sinus in two patients (5%). Residual bone height values were recorded higher in patients in whom bone-level implants were placed (37 cases; 92,5%) compared to whom received tissue-level implants (*p* = 0.045). The most common fixture shape was cylindrical (25 cases; 62,5%), which showed a higher residual bone height compared to the conical shape at the time of implant removal (*p* < 0.01). The fixture size ranged from 3.3 mm to 5 mm in diameter and 4 mm to 13 mm in length. Five were narrow-diameter implants (12,5%; diameter < 3.75 mm), and only two were short implants (5%; length < 8 mm). The residual bone height was < 4 mm (67,5%) in twenty-seven cases. Of these, fourteen were conical implant-shade (93,3% of conical implant displaced), and thirteen were cylindrical (52% of cylindrical implant displaced).

### Clinical findings

**Fig. 5:**
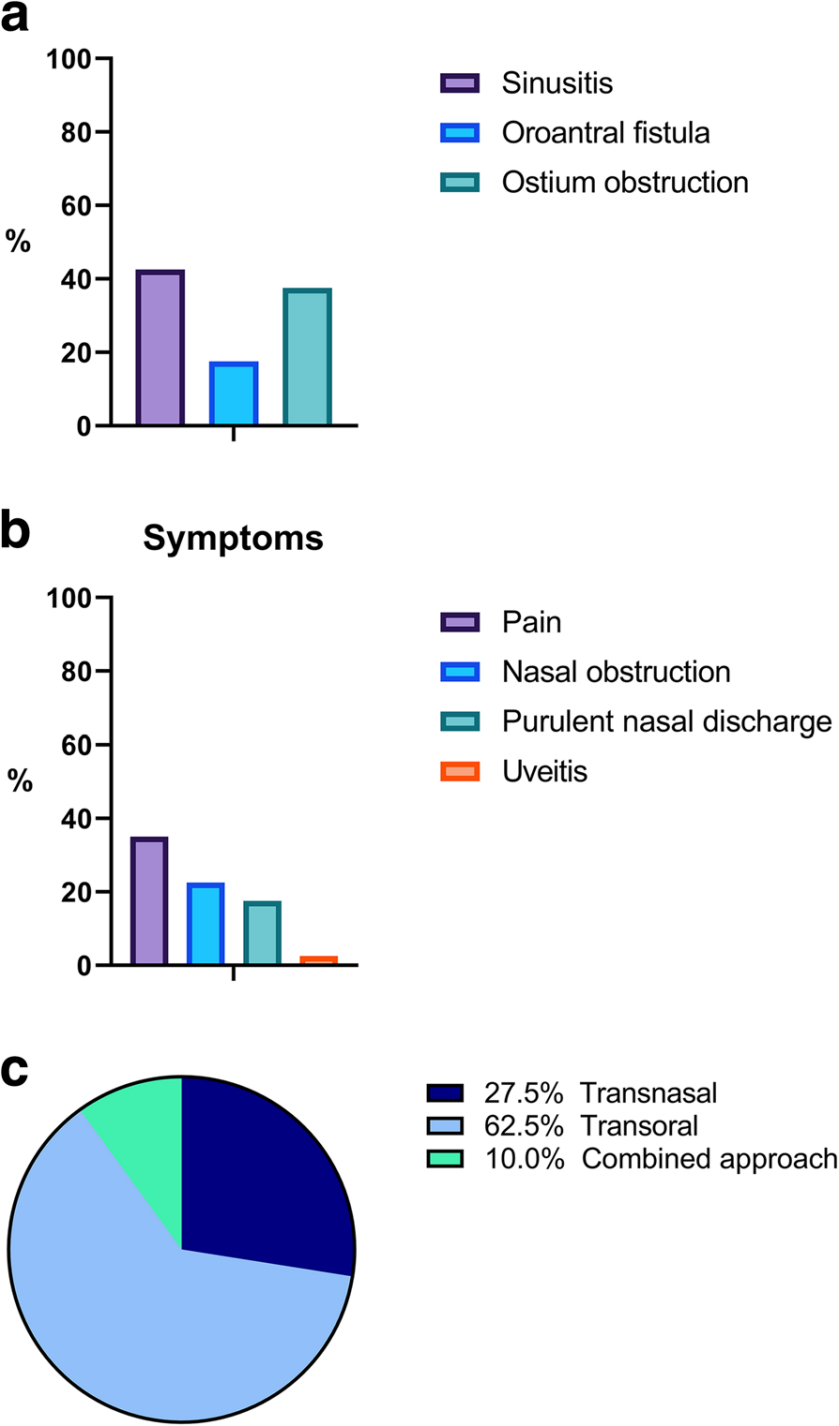
**A** Signs; **B** Symptoms; **C** Treatment

Twenty-three patients (57,5%) were asymptomatic: they were referred to the Academic Hospital immediately after implant displacement or after a casual diagnosis during a dental evaluation by OPG or CBCT scan. Seventeen patients (42,5%) sustained implant-associated chronic sinusitis (Fig. 5) associated in most cases with pain (35%), nasal obstruction (22,5%), purulent nasal discharge (17,5%). In one case, sinusitis was associated with uveitis (2,5%). Seven patients presented with OAF (17,5%). In 15 cases, an ostium obstruction was diagnosed (37,5%). Eleven patients (27,5%) were treated solely via the trans-nasal endoscopic approach, and four patients (10%) who had an associated OAF underwent surgery through a combined trans-nasal and trans-oral approach. The remaining patients underwent trans-oral surgery under local anesthesia (62,5%; Figure). All patients healed uneventfully without complications. Two patients treated with the trans-nasal approach reported a sinusitis episode successfully managed with medical therapy at the six-month follow-up visits. No patients needed surgical revision.

## Discussion

Implant displacement or migration in the maxillary sinus is a rare complication due to infections, failure to plan for surgery, or malpractice. The spread of digital technologies was supposed to reduce the incidence and the lack of diagnosis of this complication, but it is increasingly reported in the literature [11, 12]. An accurate preoperative evaluation to exclude anatomical or pathological contraindications represents the key factor in avoiding postoperative complications in the implant-prosthetic rehabilitation of the maxillary bone [13].

In cases of severe pneumatization of the maxillary sinus and thin residual alveolar bone, wrong positioning of the fixture or inaccurate prosthetic procedures can lead to implant displacement. It can occur more often in patients undergoing simultaneous sinus lift and implant placement, mainly when residual bone height is less than 3–4 mm [14, 15]. According to Galindo et al., differences in the air pressure between the maxillary sinus and nasal cavity and bone remodeling during osteointegration could cause implant displacement in case of inappropriate force application, lack of primary implant stability, unsuitable temporary denture usage, and peri-implantitis [16]. Immediate removal of implants dislocated in the maxillary sinus is strongly recommended. However, when the removal is not contextual, migrations of the fixtures can occur so that management could become complex [17].

This condition is frequently associated with OAF and sinusitis. The fixtures could migrate into ethmoid and sphenoid sinuses, orbit, and anterior cranial fossa. Other complication in implant dentistry includes ingestion or inhalation [11, 18]. Surgical approaches for removing displaced implants are trans-oral and trans-nasal. Some authors describe trans-oral and trans-nasal as two different approaches that can be used alternatively in cases where implant migration occurs [19]. The most relevant results of a literature search on the topic are summarized in Table 2.

**Table 2:** Relevant results of literature search on displacement and migration of dental implant in maxillary sinus

Reference	Cases (number of implants if different)	Patient with previous sinus lift	Patients with OAF	Patients with symptoms	Patients with radiological findings related to sinusitis	Treatments
Chiapasco et al., 2009 [11]	27	—	19	13	13	17 TO, 6 TN, 4 CA
Ridaura-Ruiz et al., 2009 [20]	9 (10)	1	—	3	6	7 TO, 2 follow-up
Matti et al., 2013 [21]	16 (17)	—	—	10	10	16 TN
Sgaramella et al., 2016 [22]	21 (24)	6	1	8	8	16 TO, 5 Caldwell-Luc
de Jong et al., 2016 [23]	14	—	9	10	8	12 TN, 1 CA, 1 expelled nasally
Manor et al., 2018 [24]	55	37 lateral, 2 crestal	37	40	38	52 TO, 1 TN, 1 CA 1 expelled nasally
Safadi et al. 2020 [25]	24 (25)	—	5	16	11	19 TN, 5 CA

*TO* Transoral approach, *TN* Transnasal approach, *CA* Combined transnasal-transoral approach, — Not reported

The authors reported a retrospective case series of forty patients with dental implant displacement in the maxillary sinus. Seventeen patients sustained implant-associated chronic sinusitis associated in most cases with pain, nasal obstruction, and purulent nasal discharge. Seven patients presented with OAF. In 15 cases, an ostium obstruction was diagnosed. Twenty-five patients underwent trans-oral surgery under local anesthesia. Eleven patients were treated solely via a trans-nasal approach, and four patients with OAF underwent surgery through a combined trans-nasal and trans-oral approach. All patients healed uneventfully without complications. Statistical analysis showed a significant difference in residual bone quantity at the moment of implant retrieval between patients that received cylindrical compared to conical implants. Specifically, the displacement of conical implants almost always occurred with less than 4 mm of residual bone, unlike those cylindrical, which also migrated with more than 4 mm of residual bone. These results could be related to an unfavorable fixture shape for cylindrical implants regarding achieving sufficient primary stability in the maxillary bone. Instead, conical implant geometry could ease the achievement of primary stability in the reduced bone amount [26]. The data analysis also highlighted a higher prevalence in the dislocation of bone level implants compared to tissue level ones in this case series. However, the number of cases examined is relatively low considering the prevalence of posterior maxilla implant-prosthetic rehabilitation. Based on literature findings and authors’ experience, implant migration could occur after surgical or prosthetic errors or infection/inflammation at the implant site. The lack of primary stability due to inadequate implant site preparation or placement in insufficient quantity or quality bone could also be possible. The bone volume was often inadequate to support the implants used in the posterior maxilla. Sinus lift or short implant placement would probably have avoided dislocation in most cases reported.

The Caldwell-Luc approach has not been used in any reported cases, as the medial inferior maxillary sinus antrostomy is not always effective in the functional restoration of the maxillary sinus [27]. Furthermore, it is associated with possible multiple complications, which in some cases require the use of vascularized bone flaps as described by Biglioli and Goisis [28].

Adequate patency of the maxillary sinus ostium is the sine qua non to recover the sinus’s ventilatory function after removing the foreign body [23]. In the case of ostium obstruction, this can be achieved with a minimally invasive procedure such as FESS. Its main advantages are the possibility of examining and treating the nasal cavity and all the paranasal sinuses, possibly involved by the infection starting from the maxillary sinus and enlarging the obstructed maxillary ostium [25].

Several authors have described a trans-nasal approach to treat sinusitis secondary to implant displacement. Matti et al. reported a series of 16 patients among a pure trans-nasal approach was used for dental implant retrieval [21].

As with any surgical procedure, FESS has associated risks and complications. Although infrequent, the most common complications are bleeding and recurrence of the disease (with persistence or worsening of sinus symptoms and facial pain). Severe complications involving the skull base and the orbit are rare (less than 0.1%). Other uncommon risks of FESS include swelling or bruising of the area around the eye, alteration of the sense of smell or taste, and change in the resonance or quality of the voice [29, 30].

In the case of OAF or alveolar infection, FESS alone may not be sufficient in treating chronic sinusitis derived from the dislocated implant and bone infection. In these cases, a combination of FESS and an intraoral approach allows the removal of foreign bodies from the sinuses with a less invasive procedure and closure of the OAF with local flaps [19]. Safadi et al., in their experience of 24 patients treated with endoscopic sinus surgery for dental implant displacement into the maxillary sinus, reported that five patients requested a combined trans-nasal and trans-oral approach because of OAF [25].

In the case of no maxillary ostium obstruction, without signs and symptoms of maxillary sinus mucosa infection, an intraoral approach with implant removal through the pre-existing implant site (or communication in the case of OAF) or after a lateral antrostomy, followed by primary closure of the access flap, may be the treatment of choice [22].

Manor et al. reported a case series of 55 patients with dental implant displacement in the maxillary sinus. In 52 cases, implants were removed through a lateral antrostomy (Caldwell-Luc like approach). Local flaps were used to treat OAF in 46 patients. Manor et al. also reported that older patients showed more cases of sinusitis and OAF and that they required more than one surgery for OAF closure and a longer hospitalization [24]. Ridaura-Ruiz et al. reported 9 cases of implant displaced in the maxillary sinus, all treated with a lateral window approach with primary wound closure because it allows good surgical access, a low rate of complications, and simple surgical technique [20]. Biglioli and Chiapasco also described the removal of 36 dental implants displaced in the maxillary sinus via an intraoral approach consisting of the creation of a bony window pedicled to the maxillary sinus membrane without complications [31].

The trans-oral approach is cost-effective because surgery can be performed under local anesthesia, and patients could be discharged immediately. However, it is not always possible to use this approach, for example, in the case of ostium obstruction [22].

**Fig. 6:**
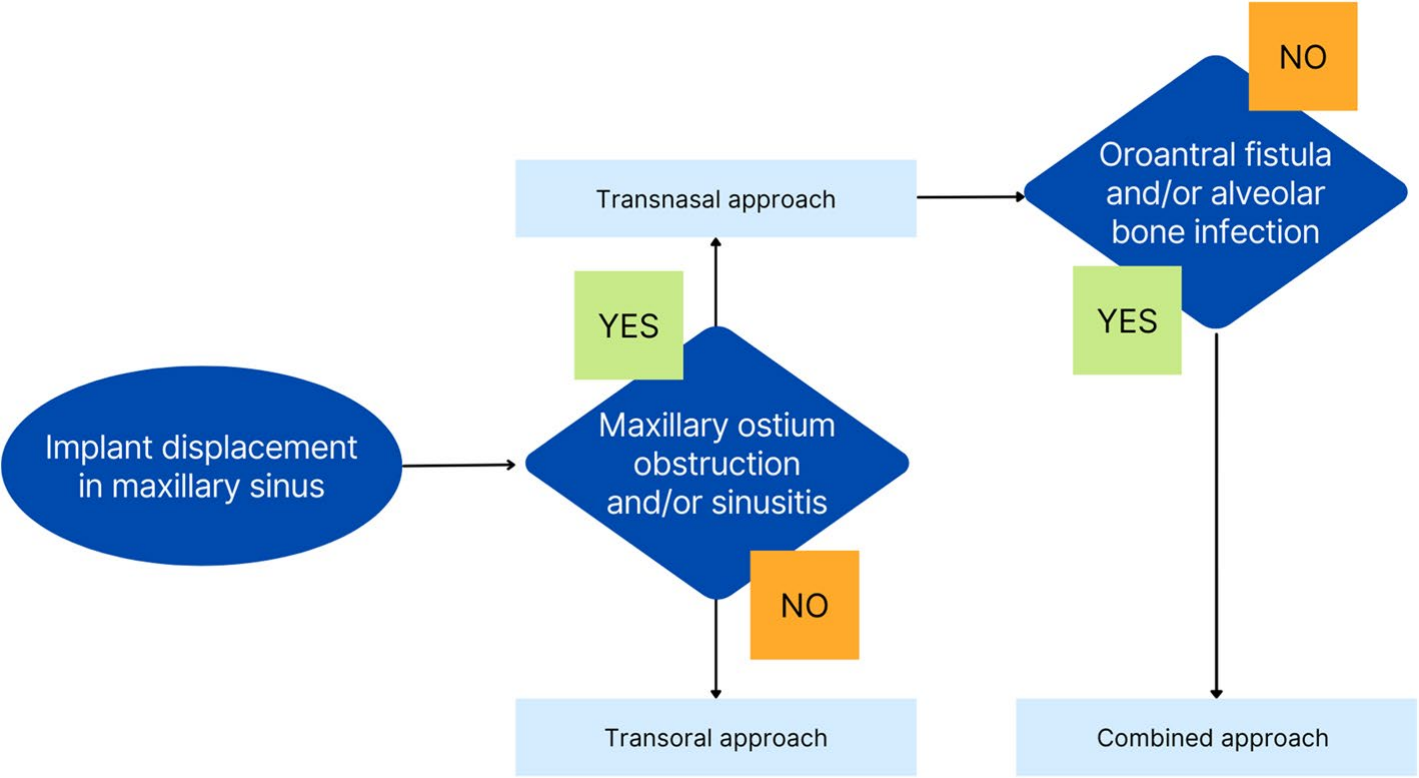
Decision tree

In conclusion, despite the limitations of this study, the flowchart for the choice of surgical treatment presented in this manuscript could be a rational proposal (Fig. 6). The use of conical implants should be preferred in the posterior atrophic maxilla. Immediate removal of the implant from the maxillary sinus is always preferable. Migration of displaced implants and sinus mucosal changes may also occur over a short period, eventually causing secondary sinusitis. Therefore, early surgical removal minimizes sinus inflammation and prevents more invasive procedures. The results presented and recent literature validate that the FESS, trans-oral approach or a combination of these procedures can be used safely to treat complications following the displacement/migration of dental implants in the maxillary sinus. Each procedure presents specific indications that must be carefully evaluated prior to treatment choice in order to optimize intervention outcomes.

## Data Availability

The data that support the findings of this study are available on request from the corresponding author.
